# Curvature-guided anisotropic noise injection for robust multimodal data processing in neuroscience and perception science

**DOI:** 10.3389/fnins.2026.1890713

**Published:** 2026-06-30

**Authors:** Yubin Wu, Huilin Liu

**Affiliations:** School of Computer Science and Engineering, Northeastern University, Shenyang, China

**Keywords:** deep learning, generalization gap, geometric anisotropic noise injection, large-batch training, multimodal learning, robust optimization

## Abstract

**Introduction:**

Large-batch training is widely used to scale multimodal neural networks that integrate heterogeneous inputs such as visual, textual, and physiological signals. However, increasing the batch size suppresses the stochastic fluctuations of mini-batch sampling, which can trap multimodal models in sharp, *modality-dominant* minima and produce a persistent generalization gap.

**Methods:**

To address this problem, we propose Geometric Anisotropic Noise Injection (GANI), a curvature-aware optimization framework inspired by information geometry and multisensory integration. GANI decouples deterministic large-batch descent from stochastic geometric exploration. It approximates local curvature through an exponential moving average of first-order gradients and injects structured anisotropic noise during parameter updates, thereby restoring the geometry-aware exploration dynamics of small-batch stochastic gradient descent with linear computational complexity.

**Results:**

Theoretical analysis shows that curvature-aligned stochasticity can accelerate escape from sharp modality-specific basins and guide parameters toward flatter regions. Experimental evaluations across multimodal benchmark settings and stress tests demonstrate that GANI reduces the generalization gap, improves convergence stability, bounds the maximum Hessian eigenvalue, and maintains stronger performance under visual noise and missing textual information than standard large-batch SGD and common adaptive optimizers.

**Discussion:**

By linking optimization geometry with multimodal representation dynamics, GANI provides an efficient and interpretable mechanism for robust heterogeneous data processing. The framework offers potential value for uncertainty-aware multisensory integration, brain-inspired perception science, and scalable multimodal learning under noisy or incomplete sensory conditions.

## 1 Introduction

Multimodal learning has become a central paradigm in modern artificial intelligence and perception science because many real-world systems must integrate heterogeneous information from multiple sources, such as images, text, audio, sensor signals, physiological measurements, and behavioral observations. In cognitive neuroscience, this ability to fuse heterogeneous inputs is closely related to *multisensory integration*, a fundamental process for understanding brain-behavior relationships. Compared with single-modality learning, multimodal models can exploit cross-modal complementarity and learn richer representations, thereby improving robustness and decision reliability in complex scenarios (Baltrusaitis et al., 2019; Liang et al., 2022; Wang et al., 2023). At the same time, the rapid growth of multimodal architectures has substantially increased model scale and training cost.

Large-batch training has therefore become an important strategy for improving computational throughput in distributed deep learning. However, as reported in previous studies, excessively large batch sizes may reduce the stochasticity of optimization and lead deep neural networks to converge to sharp minima, producing a persistent generalization gap between training and testing performance (Keskar et al., 2017; Smith et al., 2018). This tension between computational efficiency and generalization quality is particularly important for multimodal learning, where heterogeneous data distributions, modality-specific noise, and cross-modal discrepancies make robust optimization even more challenging. Without sufficient stochastic exploration, multimodal models frequently suffer from *modality dominance*—a pathological state where the optimizer rigidly overfits to a clean, dominant modality while ignoring noisier, secondary modalities. This structural brittleness prevents the model from achieving *graceful degradation* when facing sensory noise, a hallmark of robust biological perception (Lin et al., 2024).

Existing studies have attempted to alleviate this problem from several perspectives. Learning-rate scaling and layer-wise adaptive strategies, such as LARS, improve the stability of large-batch optimization by adjusting the magnitude of parameter updates (You et al., 2017), while adaptive optimizers such as Adam and RMSprop dynamically rescale gradients using first- and second-order moment estimates (Kingma and Ba, 2015). Other studies suggest that longer training, warm-up schedules, or careful learning-rate-to-batch-size ratios can partially reduce the generalization gap (Goyal et al., 2017; Hoffer et al., 2017). In parallel, recent Sharpness-Aware Minimization (SAM) methods explicitly penalize local loss sharpness and encourage convergence toward flatter minima (Foret et al., 2021; Kwon et al., 2021; Zhuang et al., 2022; Chen et al., 2023). Nevertheless, most existing solutions remain limited for robust multimodal learning in two aspects. First, methods like LARS and Adam mainly modify deterministic descent dynamics through numerical rescaling, but do not restore the beneficial stochastic exploration naturally present in small-batch SGD. Second, while SAM effectively seeks flat regions, it requires multiple forward and backward passes per step, imposing a severe computational bottleneck for large multimodal architectures. Furthermore, isotropic noise-injection methods often treat perturbations as uniform or externally imposed signals, while overlooking the fact that SGD noise itself is highly geometry-dependent and aligned with the local curvature of the loss landscape (Watanabe, 2009; Jastrzbski et al., 2017; Welling and The, 2011; Neelakantan et al., 2015). As a result, existing methods may improve optimization stability but still fail to fully reproduce the implicit geometric regularization that supports the superior generalization behavior of small-batch training.

Motivated by this observation, and drawing inspiration from the *Bayesian Brain Hypothesis*—which posits that biological neural systems actively maintain probabilistic, flexible representations to avoid overly rigid inferences under uncertainty—this paper proposes Geometric Anisotropic Noise Injection (GANI), a curvature-aware optimization framework for improving multimodal generalization under large-batch training. The central idea is that the gradient noise induced by small-batch SGD should not be regarded as simple isotropic white noise. Instead, from the perspective of information geometry and stochastic dynamics, its covariance is closely related to the Fisher information matrix and, near local minima, to the Hessian structure of the loss landscape (Jastrzbski et al., 2017). This implies that small-batch SGD naturally generates stronger fluctuations along sharp directions and weaker fluctuations along flat directions. By mathematically mimicking the *cognitive flexibility* of biological brains, this curvature-aligned stochasticity provides the precise kinetic energy required for the model to trigger a Kramers escape from sharp, modality-dominant minima, enabling the optimizer to escape narrow basins and eventually settle in flatter regions with better generalization. GANI transfers this mechanism into large-batch optimization by decoupling deterministic gradient descent from stochastic geometric exploration. Specifically, it uses large batches to preserve efficient and stable descent, while employing an exponential moving average of first-order gradients to approximate local curvature with linear O(d) computational complexity. Based on this approximation, GANI injects anisotropic structured noise during parameter updates, thereby restoring the geometry-aware exploration capability lost in large-batch training without sacrificing computational efficiency.

The main contributions of this paper are:

1. We provide a geometry-driven interpretation of the generalization gap in large-batch multimodal learning by showing that the absence of curvature-aligned stochastic fluctuations can make optimization trajectories more likely to remain in sharp and poorly generalizable minima associated with *modality dominance*.
2. We propose the GANI optimizer, which efficiently approximates local curvature through an EMA-based first-order gradient statistic and injects anisotropic noise aligned with the empirical loss geometry, offering a computationally scalable alternative to heavy sharpness-aware methods.
3. We conduct systematic experimental evaluations and geometric diagnostics, including newly introduced pathological stress tests simulating modality-specific noise and missing modalities, to demonstrate that GANI can substantially reduce the generalization gap, improve convergence stability, and guide models toward flatter minima compared with standard large-batch SGD and commonly used adaptive optimization methods.

## 2 Related work

This section reviews studies most closely related to the research problem of this paper: how to improve multimodal generalization under efficient large-batch training. Existing work has investigated multimodal representation learning, large-batch optimization, loss-landscape geometry, and stochastic noise injection from different perspectives. To explicitly address the imperative to bridge deep learning optimization and cognitive science, we review these studies by connecting computational optimization bottlenecks with principles of neuroscience data processing and brain-inspired intelligence. To clarify the methodological motivation of GANI, we organize related work into three aspects: multimodal representation learning and optimization difficulty, large-batch generalization and flat-minimum geometry, and stochastic dynamics with geometry-aware noise injection.

### 2.1 Multimodal representation learning and optimization difficulty

Multimodal learning aims to integrate heterogeneous information from multiple sources, such as images, text, audio, sensors, and physiological signals, into a unified predictive model. In the context of neuroscience and perception science, this computational paradigm mirrors the biological process of *multisensory integration*, where the brain dynamically fuses conflicting or noisy sensory inputs to form reliable cognitive representations. Recent surveys and applications have shown that multimodal fusion can improve representation richness, robustness, and decision reliability by exploiting complementary information across modalities (Baltrusaitis et al., 2019; Liang et al., 2022; Wang et al., 2023; Huang et al., 2024). In domains such as multimodal sentiment analysis and neuroscience data processing, learning systems often need to handle modality imbalance, noisy observations, temporal misalignment, and cross-modal distribution shifts (Ma et al., 2022; Han et al., 2023). These characteristics make the optimization landscape of multimodal models more complex than that of single-modality models because the final objective is shaped not only by unimodal feature extraction but also by cross-modal alignment and fusion.

Although many multimodal studies focus on architectural design, attention mechanisms, or fusion strategies, the optimization problem itself has received comparatively less attention. In practice, multimodal models are frequently trained with large batches to improve throughput, especially when multiple encoders and fusion modules increase computational cost. However, large-batch training may reduce the stochastic gradient fluctuations that help the model explore different cross-modal representation configurations. Once stochastic exploration is weakened, the model may overfit dominant modalities [a pathological state we conceptualize as *modality dominance* (Lin et al., 2024)] or converge to sharp fusion-specific minima that perform well on the training distribution but generalize poorly to unseen samples. Therefore, improving multimodal generalization requires not only better fusion architectures, but also optimization mechanisms that can actively maintain robust exploration in heterogeneous and high-dimensional parameter spaces.

### 2.2 Large-batch generalization and flat-minimum geometry

Large-batch training has become an essential technique for scaling deep neural networks because it improves hardware utilization, reduces communication overhead, and accelerates distributed training. However, Keskar et al. (2017) empirically demonstrated that large-batch methods tend to converge to sharp minima and may exhibit a clear generalization gap compared with small-batch SGD. Follow-up studies proposed learning-rate scaling, warm-up schedules, and batch-size scheduling to stabilize large-batch optimization (Smith et al., 2018; Goyal et al., 2017; Hoffer et al., 2017). You et al. (2017) further introduced LARS, which normalizes layer-wise update magnitudes and enables stable training with extremely large batches. Adaptive optimizers such as Adam also adjust update scales through gradient moment statistics (Kingma and Ba, 2015).

Despite their engineering success, these strategies mainly rescale the deterministic descent direction. They improve how parameters move along the gradient, but they do not fundamentally restore the “thermodynamic” stochastic exploration mechanism lost when the batch size becomes large. From a geometric perspective, the relationship between minima flatness and generalization has long been recognized. Hochreiter and Schmidhuber (1997) argued that flat minima are more robust to parameter perturbations and are therefore more likely to generalize well. Singular Learning Theory further indicates that deep neural networks are singular statistical models whose generalization behavior is related to algebraic-geometric quantities such as the Real Log Canonical Threshold (Watanabe, 2009). Recent Sharpness-Aware Minimization (SAM) methods explicitly penalize local loss sharpness to encourage flatter minima (Foret et al., 2021; Kwon et al., 2021; Zhuang et al., 2022; Chen et al., 2023). While theoretically sound, SAM requires adversarial gradient perturbation steps that effectively double the forward and backward computational cost. For massive multimodal architectures, this O(d) overhead per step is often computationally prohibitive. More importantly, they typically treat sharpness as an external regularization objective, rather than recovering the intrinsic curvature-dependent stochasticity of SGD. This gap motivates a more direct mechanism for guiding large-batch multimodal models toward flatter and more robust regions.

### 2.3 Stochastic dynamics and geometry-aware noise injection

Stochastic dynamics provides an important viewpoint for understanding why small-batch SGD often generalizes better than its large-batch counterpart. The noise induced by mini-batch sampling can be interpreted as a diffusion term in a stochastic differential equation, making SGD closely related to Langevin dynamics. Welling and The (2011) proposed stochastic gradient Langevin dynamics by injecting Gaussian noise into gradients for approximate Bayesian posterior sampling. Neelakantan et al. (2015) further showed that adding gradient noise during training can improve optimization and generalization in certain settings. However, most artificial noise-injection methods rely on isotropic Gaussian perturbations. Such noise is simple to implement, but it does not reflect the highly anisotropic structure of modern neural network loss landscapes (Jastrzbski et al., 2017; Zhu et al., 2018).

Recent theoretical analyses suggest that the covariance of SGD noise is closely related to the Fisher information matrix and, near local minima, to the Hessian structure of the loss landscape (Jastrzbski et al., 2017). While a growing body of recent optimization theory research highlights that gradient noise in deep neural networks often exhibits a heavy-tailed distribution (e.g., Lévy flights) rather than a strict Gaussian distribution, the fundamental consensus remains that the *anisotropy* of this noise is the crucial driver for escaping sharp minima (Zhu et al., 2018). This means that SGD noise is not merely random disturbance, but a geometry-dependent signal whose intensity varies across parameter directions. Such anisotropic diffusion can help parameters escape sharp minima and remain stable in flatter regions. Crucially, converging to these flat, highly redundant geometric regions (characterized by a low Real Log Canonical Threshold, λ) directly parallels the biological principle of *graceful degradation* in brain-inspired perception systems, where cognitive functions maintain robustness despite sensory impairment.

Motivated by this insight, the proposed GANI framework differs from conventional noise-injection methods by synthesizing structured noise aligned with empirical curvature. By estimating curvature through an exponential moving average of first-order gradients, GANI provides an efficient and scalable way to recover the geometry-aware exploration of small-batch SGD within large-batch multimodal training. In this sense, GANI directly targets the unresolved problem shared by prior work: preserving large-batch efficiency while restoring the curvature-sensitive stochasticity required for robust multimodal generalization.

## 3 Geometric selectivity in multimodal SGD

### 3.1 The geometric nature of stochastic noise in multimodal training

In standard optimization analyses, the gradient noise caused by mini-batch sampling is often simplified as isotropic Gaussian white noise. This simplification is particularly problematic for multimodal learning, where parameters are distributed across modality-specific encoders, cross-modal alignment layers, and fusion modules. Different modalities may generate gradients with substantially different magnitudes, noise levels, and curvature structures. Therefore, to understand why small-batch SGD can improve multimodal generalization, it is necessary to reveal the geometry of stochastic gradient noise rather than treating it as uniform disturbance.

Assume a multimodal training dataset $Z=\left\{\left(x^{\left(1\right)},x^{\left(2\right)},\ldots ,x^{\left(M\right)},y\right)\right\}$ with *N* samples, where *M* denotes the number of modalities. A multimodal neural network with parameters θ∈ℝ^*d*^ consists of modality-specific representation functions and a fusion function for prediction. For a given multimodal sample *z* = (*x*^(1)^, *x*^(2)^, …, *x*^(*M*)^, *y*), we define the single-sample Negative Log-Likelihood (NLL) loss as ℓ(*z*; θ) = −log*p*(*y*|*x*^(1)^, *x*^(2)^, …, *x*^(*M*)^; θ).

In the *t*-th iteration of training, SGD randomly draws a mini-batch of data $B_{t}$ of size *B* from the dataset. The mini-batch gradient ĝ_*B*_(θ_*t*_) calculated by SGD can be decomposed into a deterministic full gradient ∇*L*(θ_*t*_) and a zero-mean random noise vector ξ_*t*_:


$$\begin{array}{l} {ĝ}_{B}\left(\theta_{t}\right)=\nabla L\left(\theta_{t}\right)+\xi_{t} \end{array}$$ (S1)

The core issue here is to determine the statistical characteristics of the noise term ξ_*t*_. According to the Central Limit Theorem (CLT), when the batch size *B* is moderate (satisfying 1≪*B*≪*N*), the random vector ξ_*t*_ asymptotically follows a multivariate Gaussian distribution $N\left(0,\Sigma_{B}\left(\theta \right)\right)$ (Jastrzbski et al., 2017). The covariance matrix of the noise Σ_*B*_(θ) is entirely determined by the covariance matrix *C*(θ) of the single-sample gradients and scales inversely proportional to the batch size *B*:


$$\begin{array}{l} \Sigma_{B}\left(\theta \right)=\frac{1}{B}C\left(\theta \right)=\frac{1}{B}{𝔼}_{z\sim Z}\left[\left(\nabla \ell \left(z;\theta \right)-\nabla L\left(\theta \right)\right){\left(\nabla \ell \left(z;\theta \right)-\nabla L\left(\theta \right)\right)}^{T}\right] \end{array}$$ (S2)

In the later stages of model training, when the parameter approaches a certain local minimum, the full gradient approaches zero, i.e., ∇*L*(θ)≈0. At this time, the expression of the covariance matrix above can be simplified to the uncentered second moment of the single-sample gradients:


$$\begin{array}{l} C\left(\theta \right)\approx {𝔼}_{x,y}\left[\nabla_{\theta }\ell \left(z;\theta \right)\nabla_{\theta }\ell {\left(z;\theta \right)}^{T}\right] \end{array}$$ (S3)

Substituting ℓ(*z*; θ) = −log*p*(*y*|*x*; θ) into the above equation, we obtain:


$$\begin{array}{l} C\left(\theta \right)\approx {𝔼}_{x,y}\left[\nabla_{\theta }\log p\left(y|x;\theta \right)\nabla_{\theta }\log p{\left(y|x;\theta \right)}^{T}\right] \end{array}$$ (S4)

It is worth noting that the expression of this second moment is exactly the classical definition of the Fisher Information Matrix (FIM) in information geometry, usually denoted as *F*(θ). From this, we have derived a fundamental conclusion regarding the nature of SGD noise: the gradient noise covariance matrix of SGD is not a scalar multiple of the identity matrix, but is strictly proportional to the Fisher Information Matrix of the parameter space, and is inversely proportional to the batch size *B*:


$$\begin{array}{l} \Sigma_{B}\left(\theta \right)\approx \frac{1}{B}F\left(\theta \right) \end{array}$$ (S5)

Together, Equations S1–S5 establish the transition from the mini-batch gradient decomposition to the Fisher-scaled anisotropic covariance of SGD noise.

This derivation indicates that the noise generated by SGD possesses strong Anisotropy. In the context of multimodal learning, this geometric anisotropy acts as an endogenous geometric sensor: it naturally differentiates between high-curvature directions (typically encoding overconfident, isolated unimodal features) and low-curvature directions (governing delicate cross-modal alignment and fusion). A uniform noise assumption would completely ignore these functional differences.

### 3.2 Fisher-Hessian identity and anisotropic diffusion

To transform the statistical characteristics of multimodal gradient noise into geometric topographical features of the loss landscape, we introduce a core theorem in information geometry: the Fisher-Hessian Identity. This connection is important because the local curvature of a multimodal objective reflects not only class-discriminative learning, but also the stability of cross-modal alignment and the reliability of fused representations.

Near a minimum point θ^*^, the geometric curvature of the loss function is characterized by its second derivative, namely the Hessian matrix *H*(θ) = ∇^2^*L*(θ). For models using Negative Log-Likelihood (NLL) as the loss function, if the model possesses sufficient expressiveness to truly fit the data distribution (i.e., satisfying the Realizable Assumption), then under regular conditions, the expected Hessian matrix of the loss function is asymptotically equivalent to the Fisher Information Matrix:


$$\begin{array}{l} 𝔼\left[H\left(\theta^{*}\right)\right]=F\left(\theta^{*}\right) \end{array}$$ (S6)

This identity establishes a profound mathematical dual relationship: the curvature (Hessian) of the loss function surface is, in expectation, equal to the variance (Fisher) of the first-order gradient itself. Combining the conclusion $\Sigma_{B}\left(\theta \right)\approx \frac{1}{B}F\left(\theta \right)$ reached in Section 3.1, we can directly link the noise covariance matrix of SGD to the local curvature of the loss function:


$$\begin{array}{l} \Sigma_{B}\left(\theta \right)\approx \frac{1}{B}H\left(\theta \right) \end{array}$$ (S7)

In actual parameter updates, the gradient term also needs to be multiplied by the learning rate η. According to the linear scaling property of covariance, the noise covariance matrix Σ_*inject*_ actually borne by the parameters in a single step update is:


$$\begin{array}{l} \Sigma_{inject}\approx \frac{\eta^{2}}{B}H\left(\theta \right) \end{array}$$ (S8)

Equations S7 and S8 therefore translate the Fisher-noise covariance into Hessian-aligned curvature and the actual update-scale covariance borne by the parameters.

To more intuitively analyze the evolutionary trajectory of parameters in multi-step iterations, we can approximate the discrete update equation of SGD as a Stochastic Differential Equation (SDE) in the parameter space at the continuous time limit (i.e., when η → 0). At this time, the evolution of SGD manifests as Langevin Dynamics on a Riemannian manifold:


$$\begin{array}{l} d\theta_{t}=-\nabla L\left(\theta_{t}\right)dt+\frac{\eta }{B}\sqrt{H\left(\theta_{t}\right)}dW_{t} \end{array}$$ (S9)

Where *dW*_*t*_ is standard multi-dimensional Brownian motion. In this continuous dynamical equation, the diffusion term containing *dW*_*t*_ (i.e., the noise term) clearly demonstrates the Anisotropic Diffusion feature of SGD noise. The distribution of the noise is not a uniform sphere but is modulated by the Hessian matrix $\sqrt{H\left(\theta_{t}\right)}$. This produces extremely important dynamic consequences:

1. **In steep directions:** When the curvature of the loss function in a certain direction is immense (extremely large Hessian eigenvalue), the diffusion term coefficient is significantly amplified, causing the parameter to experience extremely violent random oscillations in that direction.
2. **In flat directions:** When the curvature of the loss function in a certain direction is minimal (Hessian eigenvalue approaching zero), the diffusion term coefficient decreases accordingly, the noise almost vanishes, and parameter evolution is primarily dominated by the deterministic descent term −∇*L*(θ_*t*_)*dt*.

This mathematical mechanism where “noise intensity is strictly positively correlated with local curvature” proves that small-batch SGD possesses an inherent geometric selection tendency. Physically, in a multimodal architecture, an extremely large Hessian eigenvalue typically indicates that the model is severely overfitting a single dominant modality (forming a steep, brittle ravine). Equation S9 dictates that the system will spontaneously generate violent random oscillations along this specific direction, preventing the network from settling into *modality dominance*. This conclusion provides the theoretical basis for analyzing how anisotropic diffusion supports escape from sharp multimodal minima.

### 3.3 Local spectral decomposition and Kramers Escape in multimodal landscapes

The Riemannian manifold Langevin dynamic equation derived in the previous section indicates that the structured noise injected by SGD strictly depends on the local curvature of the parameter space. To quantitatively characterize mathematically how this anisotropic diffusion helps the model jump out of local suboptimal solutions, we introduce the Kramers Escape Rate Theory from statistical physics, combined with the local spectral decomposition of the loss landscape for rigorous derivation.

Assume the optimization process brings the parameter into the neighborhood of a local minimum θ^*^. We perform a second-order Taylor expansion on the loss function near this point to establish a local quadratic approximation:


$$\begin{array}{l} L\left(\theta \right)\approx L\left(\theta^{*}\right)+\frac{1}{2}{\left(\theta -\theta^{*}\right)}^{T}H\left(\theta^{*}\right)\left(\theta -\theta^{*}\right) \end{array}$$ (S10)

At this time, the deterministic gradient driving parameter evolution exhibits a linear feature, i.e., ∇*L*(θ)≈*H*(θ^*^)(θ−θ^*^). Since the Hessian matrix *H*(θ^*^) is real-symmetric and positive semi-definite, we can perform spectral decomposition (eigenvalue decomposition) on it:


$$\begin{array}{l} H\left(\theta^{*}\right)=Q\Lambda Q^{T} \end{array}$$ (S11)

Where *Q* is an orthogonal matrix composed of eigenvectors, representing the principal curvature directions of the loss surface; Λ = diag(λ_1_, λ_2_, …, λ_*d*_) is the corresponding eigenvalue matrix, and λ_*i*_ characterizes the curvature magnitude in the *i*-th principal direction. To strip away the coupling relationships between various parameter dimensions in high-dimensional space, we introduce an orthogonal coordinate transformation, setting ϕ = *Q*^*T*^(θ−θ^*^). Under the orthogonal basis, the multi-dimensional stochastic differential equation is completely decoupled into *d* independent one-dimensional stochastic differential equations. For the *i*-th principal curvature direction ϕ_*i*_, its evolutionary dynamics satisfy:


$$\begin{array}{l} d\phi_{i}=-\lambda_{i}\phi_{i}dt+\sqrt{\frac{\eta }{B}\lambda_{i}}dW_{i} \end{array}$$ (S12)

This decoupled equation clearly shows the dynamic competitive relationship on a single dimension:

1. **Drift term** **(−λ_*i*_ϕ_*i*_*dt*):** Proportional to curvature λ_*i*_, representing the deterministic restoring force of the loss surface attempting to pull the parameter back to the bottom of the minimum.
2. **Diffusion term** $\left(\sqrt{\frac{\eta }{B}\lambda_{i}}dW_{i}\right)$: Represents the random fluctuations injected by SGD in that direction. Crucially, its fluctuation amplitude is similarly positively amplified by the curvature λ_*i*_.

Now, we examine the average time required for the parameter to escape the current minimum by crossing the potential barrier (i.e., crossing the saddle point) along the *i*-th direction. Here we need to introduce Kramers escape theory. Its core formula (the Arrhenius equation) is very simple; in the weak noise limit, the escape time τ_*i*_ exponentially depends on the ratio of the barrier height Δ*L*_*i*_ to the effective diffusion coefficient *D*_*i*_:


$$\begin{array}{l} \tau_{i}\propto \exp\left(\frac{\Delta L_{i}}{D_{i}}\right) \end{array}$$ (S13)

In the one-dimensional SDE above, the effective diffusion coefficient (i.e., equivalent thermodynamic temperature) can be derived from half the variance of the diffusion term, i.e., $D_{i}=\frac{1}{2}\frac{\eta }{B}\lambda_{i}$. Meanwhile, assuming the characteristic width of the barrier in that direction from the bottom of the minimum is *R*_*i*_, the barrier height under the local quadratic approximation can be represented as $\Delta L_{i}\approx \frac{1}{2}\lambda_{i}R_{i}^{2}$. Substituting the barrier height and diffusion coefficient into the escape time formula, we obtain the core mathematical expression for the geometric selectivity of SGD:


$$\begin{array}{l} \tau_{i}\propto \exp\left(\frac{BR_{i}^{2}}{\eta }\right) \end{array}$$ (S14)

Equations S10–S14 summarize the local quadratic approximation, Hessian spectral decomposition, one-dimensional stochastic dynamics, Kramers escape time, and curvature-cancelled escape exponent.

This result reveals a profound counter-intuitive phenomenon: **In the escape exponent of SGD, the local curvature** **λ_*i*_** **is completely canceled out**. If the optimization algorithm uses isotropic uniform Gaussian white noise, the escape time would be proportional to $\exp\left(\lambda_{i}R_{i}^{2}/2D\right)$, which means that the greater the curvature λ_*i*_ in a sharp direction, the harder the barrier is to overcome, and the parameters will highly likely be deadlocked in sharp minima. However, in small-batch SGD, this constraint is completely shattered. While sharp minima generate massive gradient restoring forces, they also proportionally amplify the variance of the local diffusion term. This endogenous “adaptive oscillation” endows the parameters with sufficient kinetic energy needed to overcome high-curvature barriers. Therefore, sharp directions cannot form effective dynamic capture traps, and SGD will continuously diffuse in the parameter space until it encounters flat regions where curvature is generally small and the escape time is sufficiently long.

From a perception science perspective, this Kramers escape mechanism bears a striking resemblance to *cognitive flexibility* in biological multisensory integration. When the brain receives conflicting or overly noisy input from one sensory channel (analogous to a sharp, unstable parameter space), it avoids locking into a rigid, deterministic inference. Instead, it utilizes probabilistic stochasticity to explore alternative neural pathways, eventually settling on a more stable, cross-modally fused perception.

### 3.4 Singular learning theory, entropic force, and multimodal flatness

The Kramers escape mechanism explains from a micro-dynamic level why small-batch SGD struggles to reside in local high-curvature regions. However, to fully describe the macroscopic convergence distribution of the system after long-term evolution, we need to introduce the thermodynamic equilibrium state theory from statistical physics and Singular Learning Theory (SLT) proposed by Sumio Watanabe (Watanabe, 2009).

Under a small learning rate (η → 0) and sufficiently long evolution time, the SGD system driven by Riemannian manifold Langevin dynamics will gradually approach a Stationary Distribution. This distribution can be derived from the Fokker-Planck Equation and is asymptotically equivalent to the Gibbs Distribution in statistical mechanics:


$$\begin{array}{l} P_{ss}\left(\theta \right)\propto \exp\left(-\frac{L_{S}\left(\theta \right)}{T_{eff}}\right) \end{array}$$ (S15)

Where the equivalent thermodynamic temperature of the system is $T_{eff}\propto \frac{\eta }{B}$.

When assessing the generalization capability of a model, our focus is not on a single isolated point θ^*^ in the parameter space, but a macroscopic region Ω containing that minimum. The posterior Probability Mass of this region is determined by the partition function (integral volume) of that region:


$$\begin{array}{l} P\left(\Omega \right)=\underset{\theta \in \Omega }{\int }P_{ss}\left(\theta \right)d\theta \approx \exp\left(-\frac{L_{min}}{T_{eff}}\right)\text{Volume}\left(\Omega \right) \end{array}$$ (S16)

According to the principles of statistical mechanics, the macroscopic objective of system evolution is to minimize its free energy $F=E-T_{eff}S$. Here, the energy *E* corresponds to the empirical loss *L*_*min*_ of the local minimum, while the entropy *S* corresponds to the logarithm of the geometric volume of the parameter neighborhood where the minimum is located, i.e., *S*∝ln(Volume(Ω)).

From the perspective of information geometry, employing early concatenation is not an arbitrary engineering choice, but a controlled topological setup. In this concatenated parameter space, the off-diagonal blocks of the Fisher Information Matrix (FIM) explicitly encode the second moments of the joint distribution between visual and textual features, providing a rigorous testbed to evaluate GANI's capacity to navigate highly entangled, heterogeneous multimodal manifolds.

When Large Batch is used for training, the equivalent temperature *T*_*eff*_ → 0, the influence of the entropy term *T*_*eff*_*S* is greatly weakened, and the optimization process degrades into pure energy minimization (searching for deep valleys). But in the high-temperature environment induced by Small Batch, *T*_*eff*_ significantly increases, and the system evolution is dominated by a powerful Entropic Force. To minimize free energy, the system would rather tolerate a slight increase in empirical loss *E* than avoid irreversibly migrating to the region with the largest volume (maximum entropy) in the parameter space.

For traditional Regular statistical models, the volume of the minimum neighborhood can be easily calculated through multivariate Gaussian integration (Laplace approximation). However, deep and multimodal neural networks are inherently singular. Due to structural redundancy such as node permutation symmetry, dead neurons, linear dependence, modality redundancy, and many-to-one fusion mappings, the mapping from the parameter space to the function space is highly non-identifiable. To provide an intuitive illustrative example, consider the hidden nodes in a simple multilayer perceptron. The permutation of these hidden nodes (swapping their positions) or the existence of “dead” neurons (where all outgoing weights are scaled to zero) creates continuous geometric valleys of identical empirical loss, rather than isolated minimum points. Algebraically, this structural redundancy causes the Fisher Information Matrix to possess numerous zero eigenvalues. This degeneracy strictly drives the theoretical bound of λ well below the regular model bound of *d*/2, geometrically expanding the volume of the flat minimum basin. This causes its Fisher Information Matrix *F*(θ) to severely degenerate at the minimum (containing a massive number of zero eigenvalues), rendering the classic Laplace approximation completely ineffective.

To rigorously quantify the integral volume in such singular models, Singular Learning Theory introduces Hironaka's Resolution of Singularities from algebraic geometry. Theory proves that for a training set containing *n* samples, the asymptotic expansion of its local partition function (integral volume) strictly follows:


$$\begin{array}{l} Z_{n}\propto n^{-\lambda }{\left(\log n\right)}^{m-1} \end{array}$$ (S17)

Equations S15–S17 connect the steady-state distribution, the probability mass of a basin, and the RLCT-controlled asymptotic volume that explains flat-minimum robustness.

Where the exponent λ is called the Real Log Canonical Threshold (RLCT), and *m* is the multiplicity. λ is an algebraic invariant of the local geometric structure of the parameter space, which precisely characterizes the flatness and degree of degeneracy near the minimum:

1. For regular models, λ = *d*/2 (*d* is the parameter dimension).
2. For singular models such as deep neural networks, due to severe parameter redundancy in flat minimum regions, λ≪*d*/2.

In the asymptotic expansion of the partition function, λ is located in the exponent position. This means that even a minuscule decrease in λ will cause the volume of the minimum region in the parameter space to exhibit a polynomial or even exponential explosion relative to the sample size *n*.

In the context of brain-inspired multimodal perception, this geometric pursuit of low-λ (highly redundant) regions provides a mathematically rigorous foundation for *graceful degradation* (Lau et al., 2023). A biological neural network exhibits graceful degradation when the loss of a specific sensory input (e.g., visual impairment) does not lead to catastrophic cognitive failure, because redundant cross-modal synaptic connections sustain the system's reasoning. By forcing the artificial multimodal network to converge into geometrically redundant, low-λ flat minima, the anisotropic noise injection mechanism directly encodes this biological resilience into the parameter space, serving as a highly efficient implicit Bayesian regularization against sensory corruption.

## 4 Geometric anisotropic noise injection for multimodal generalization

### 4.1 Motivation and core design philosophy

In large-scale multimodal deep learning, training often involves multiple encoders and fusion layers, which substantially increases computational cost. To maximize the throughput of GPU clusters and reduce communication overhead between nodes, extremely large batch sizes (e.g., 4,096 or 8,192) are commonly adopted. However, this engineering optimization may conflict with the generalization capability of multimodal models because it suppresses the stochastic fluctuations needed to explore robust cross-modal representations.

#### 4.1.1 The theoretical dilemma of large-batch training

As we proved in the third part, the gradient noise covariance matrix of SGD is inversely proportional to the batch size *B*, i.e., $\Sigma_{B}\propto \frac{1}{B}H\left(\theta \right)$. When training with a large batch *B*_*large*_, the covariance matrix Σ_*B*_ approaches zero. From the perspective of statistical dynamics, this means the equivalent thermodynamic temperature of the system $T_{eff}\propto \frac{\eta }{B_{large}}$ sharply drops to near absolute zero. Lacking sufficient diffusion noise (thermal fluctuations):

1. Parameters lose the kinetic energy required to trigger the Kramers escape mechanism and become prone to being trapped in sharp minima, which may correspond to brittle modality-specific or fusion-specific solutions.
2. The entropic force mechanism in Singular Learning Theory (SLT) weakens, so system evolution degenerates toward pure empirical loss minimization and loses the ability to seek large-volume flat regions with a low Real Log Canonical Threshold (RLCT, λ) that support robust multimodal generalization.

#### 4.1.2 Core design philosophy: decoupling gradient descent and geometric exploration

An inherent flaw of the standard SGD algorithm is that it forcefully binds the “deterministic descent direction” and the “stochastic geometric exploration” to the same hyperparameter—batch size *B*. Decreasing *B* improves generalization but sacrifices computational speed; increasing *B* boosts speed but sacrifices generalization. To overcome this contradiction, this section proposes a brand-new optimization algorithm framework—Geometric Anisotropic Noise Injection (GANI). The core design concept of GANI is “Decoupling,” which breaks down the parameter update into two independently controllable processes:

1. Main body descent (utilizing large batches to guarantee efficiency): Using an extremely large physical batch *B*_*large*_ for multimodal forward and backward propagation. This fully utilizes hardware parallelism and calculates a low-variance empirical gradient ∇*L*_*S*_(θ_*t*_), enabling modality encoders and fusion modules to reduce loss efficiently and stably.
2. Geometric exploration (injecting structured noise artificially): We no longer rely solely on the natural randomness generated by small-batch data sampling. Instead, GANI injects synthesized noise ξ_*t*_ with covariance Σ_*inject*_ during each parameter update, so that modality-specific and cross-modal parameters receive curvature-aware exploratory perturbations.

#### 4.1.3 Mathematical derivation of synthesized noise

To enable large-batch multimodal training to approximate the generalization behavior of small-batch SGD, we need to calculate the covariance magnitude required for the artificially injected noise ξ_*t*_. Assume the generalization capability we expect the model to exhibit corresponds to a smaller target batch size *B*_*target*_ (e.g., 64 or 128). The noise covariance of small-batch SGD in an ideal scenario is:


$$\begin{array}{l} \Sigma_{target}\approx \frac{\eta^{2}}{B_{target}}H\left(\theta \right) \end{array}$$ (S18)

While the faint noise covariance inherently brought by our current large-batch calculation is:


$$\begin{array}{l} \Sigma_{large}\approx \frac{\eta^{2}}{B_{large}}H\left(\theta \right) \end{array}$$ (S19)

Since the covariance of independent random noise is additive, our artificially injected noise covariance Σ_*inject*_ must exactly bridge the gap between the two:


$$\begin{array}{l} \Sigma_{inject}=\Sigma_{target}-\Sigma_{large}=\eta^{2}\left(\frac{1}{B_{target}}-\frac{1}{B_{large}}\right)H\left(\theta \right) \end{array}$$ (S20)

Equations S18–S20 define the target small-batch covariance, the retained large-batch covariance, and the missing covariance that GANI must inject.

This derivation indicates that GANI should not inject uniform isotropic white noise. Instead, it must inject anisotropic structured noise aligned with local curvature. In this way, without sacrificing large-batch speed, GANI approximates the thermodynamic exploration behavior of small-batch SGD at the algorithmic level, reactivates curvature-sensitive escape dynamics, and guides multimodal parameters toward flatter minima with stronger robustness to modality noise and distribution shifts.

### 4.2 Empirical curvature estimation via EMA

According to the theoretical derivations in the third part, for the artificially injected random noise ξ_*t*_ to align with the local geometric features of a multimodal loss landscape, the algorithm must acquire or approximate the Hessian matrix *H*(θ_*t*_) at the model's current position in real-time. However, in modern deep neural networks, the number of parameters *d* often reaches tens of millions or even hundreds of billions. In this context, the storage space complexity of the complete Hessian matrix *H*(θ)∈ℝ^*d*×*d*^ is as high as $O\left(d^{2}\right)$, and the time complexity of performing eigenvalue decomposition or matrix element sampling on it reaches $O\left(d^{3}\right)$. Such high-order computational and memory overheads are completely unbearable in actual engineering.

#### 4.2.1 Fisher diagonal approximation and linear complexity transformation

To reduce the computational and space complexity of the algorithm to a linear level of O(d) for both space and time, GANI adopts the Diagonal Approximation commonly used in statistical physics and optimization theory. We ignore the cross second derivatives between different parameter dimensions (i.e., the off-diagonal elements of the Hessian matrix) and focus solely on the local curvature of each parameter axis itself. According to the Fisher-Hessian Identity established in the second part, near a local minimum and under the regular conditions of NLL loss, the diagonal elements of the expected Hessian matrix are asymptotically equivalent to the diagonal elements of the Fisher Information Matrix. For the *i*-th parameter θ_*i*_, its local curvature *H*_*ii*_(θ_*t*_) can be empirically estimated through the uncentered second moment of the single-sample gradient at the current parameter point:


$$\begin{array}{l} H_{ii}\left(\theta_{t}\right)\approx F_{ii}\left(\theta_{t}\right)=E_{z\sim D}\left[{\left(\frac{\partial \ell \left(z;\theta_{t}\right)}{\partial \theta_{t,i}}\right)}^{2}\right] \end{array}$$ (S21)

This mathematical relationship brings fundamental simplification to curvature calculation: it transforms the highly complex second-derivative information into a statistic that only requires the square of the first-order gradient.

#### 4.2.2 Introduction of exponential moving average and dynamic terrain tracking

Although the square of the first-order gradient can be easily obtained through the automatic differentiation mechanisms of mainstream deep learning frameworks, in actual training, we cannot directly calculate the expectation *E*_*z*~*D*_[·] over the true data distribution *D* containing infinite samples. Even settling for the second best—calculating the gradient variance over the entire training set at each iteration—would incur computational delays (O(Nd)) that completely negate the parallel acceleration advantages of large-batch training.

To resolve this expectation estimation problem of streaming data, GANI introduces the Exponential Moving Average (EMA) mechanism for the square of the gradients. Inside the optimizer, we maintain a persistent state vector *v*∈ℝ^*d*^ for each model parameter. During the *t*-th iteration, utilizing the calculated large-batch average gradient *g*_*t*_, we perform online updates on the state vector *v*_*t*_ according to the following recursive formula:


$$\begin{array}{l} v_{t}=\beta v_{t-1}+\left(1-\beta \right)\left(g_{t}⊙g_{t}\right) \end{array}$$ (S22)

Where ⊙ represents the Hadamard Product, i.e., element-wise multiplication of vectors; β∈[0, 1) is a hyperparameter representing the forgetting factor (or decay rate) of historical information, typically configured as 0.99 or 0.999 in engineering practice.

#### 4.2.3 Dynamic analysis of the update mechanism

The recursive update formula is mathematically equivalent to an exponentially weighted traceback of the squares of historical gradients:


$$\begin{array}{l} v_{t}=\left(1-\beta \right)\overset{t}{\underset{k=1}{\sum }}\beta^{t-k}\left(g_{k}⊙g_{k}\right) \end{array}$$ (S23)

Equations S21–S23 specify the diagonal Fisher curvature estimate, its EMA update, and its equivalent exponentially weighted historical form.

The parameter β strictly determines the “memory half-life” of the algorithm regarding the local geometric topology of the loss surface (approximately $\frac{1}{1-\beta }$ iterations). This online update mechanism enables the state vector *v*_*t*_ to extremely sensitively and smoothly track the dynamic terrain changes when parameters wander on the non-convex loss surface:

1. When parameters traverse steep regions: If the loss changes extremely drastically in dimension *i*, the absolute values of the gradient *g*_*t, i*_ continuously calculated in that dimension will remain high. Through squared accumulation, the state variable *v*_*t, i*_ will rapidly elevate, accurately reflecting the physical fact of high curvature in that direction.
2. When parameters enter flat basins: As the parameters gradually approach gentle regions with generally low curvature, the numerical values across all dimensions of the gradient vector *g*_*t*_ quickly converge to zero. At this time, since the newly pouring gradient squared terms (*g*_*t*_⊙*g*_*t*_) → 0, the state variable *v*_*t*_ will decay exponentially according to the proportion β, objectively manifesting as a smooth decline in the local curvature estimate.

By introducing this EMA-based curvature estimation method using first-order gradients, GANI bypasses the hardware cost of directly constructing a high-dimensional Hessian matrix for multimodal networks. With extremely low computational and memory costs, it captures the local curvature features of the parameter manifold at every moment of training, laying the engineering feasibility foundation for the precise synthesis of anisotropic structured noise.

### 4.3 Synthesis of structured noise and algorithm implementation

After obtaining the local curvature estimates *v*_*t*_ for all parameter dimensions via EMA, the next step of GANI is to synthesize structured noise and inject it into the multimodal parameter update process. Assume the system currently uses a physical large batch size of *B*_*large*_, and the target small batch size corresponding to the desired generalization performance is *B*_*target*_. According to the theoretical derivation in Section 4.1, the artificially supplemented noise covariance matrix we need is:


$$\begin{array}{l} \Sigma_{inject}=\eta^{2}\left(\frac{1}{B_{target}}-\frac{1}{B_{large}}\right)H\left(\theta_{t}\right) \end{array}$$ (S24)

To accurately and efficiently inject this variance in algorithm implementation, we first define a global noise scaling coefficient γ that is independent of data and parameters:


$$\begin{array}{l} \gamma =\sqrt{\frac{1}{B_{target}}-\frac{1}{B_{large}}} \end{array}$$ (S25)

In the *t*-th iteration, for each dimension in the parameter space, the algorithm independently samples Gaussian white noise $\epsilon_{t}\sim N\left(0,I\right)$ from a standard normal distribution. Subsequently, it utilizes the local curvature estimate *v*_*t*_ to conduct anisotropic modulation on the white noise, synthesizing the final injected noise ξ_*t*_:


$$\begin{array}{l} \xi_{t}=\eta_{t}\cdot \gamma \cdot \sqrt{v_{t}+\epsilon_{num}}⊙\epsilon_{t} \end{array}$$ (S26)

In this formula, ⊙ denotes element-wise multiplication. ϵ_*num*_ is a minuscule positive real constant (e.g., 10^−8^), whose role is to guarantee numerical stability and prevent computational anomalies that might be triggered when parameters are in absolutely flat regions (i.e., local empirical curvature is zero).

Crucially, it should be noted that the injected geometric noise ξ_*t*_ is explicitly proportional to the current learning rate η_*t*_. By coupling GANI with a Cosine Annealing learning rate schedule, the exploration noise naturally and smoothly anneals to zero at the end of training, allowing the multimodal model to converge stably to a stationary point.

Finally, parameter evolution is completed by the superposition of deterministic large-batch gradient descent and the artificially synthesized structured geometric noise:


$$\begin{array}{l} \theta_{t+1}=\theta_{t}-\eta_{t}\cdot g_{t}+\xi_{t} \end{array}$$ (S27)

S24–S27 define the practical injected covariance, global scaling coefficient, synthesized geometric noise, and final GANI parameter update.

The complete execution flow of the GANI optimization algorithm is shown in the pseudocode below. To prevent early training divergence caused by chaotic gradients, GANI strictly intervenes only after a designated Warm-up phase.

Algorithm 1 Geometric Anisotropic Noise Injection Optimizer (GANI) 
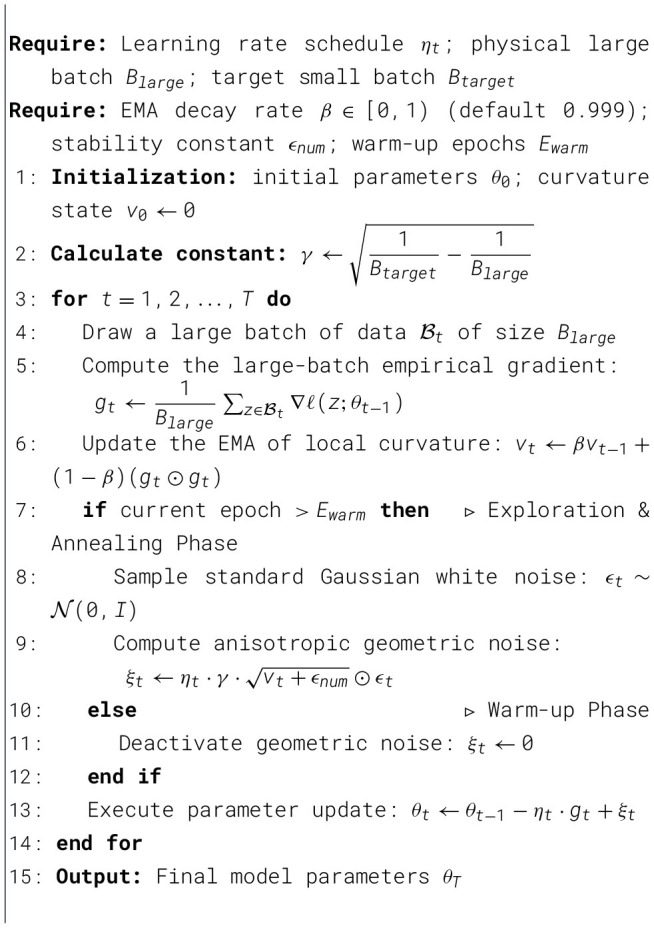

### 4.4 Accurate curvature estimation via gradient accumulation

In practical large-scale multimodal training, restricted by the VRAM capacity of a single-node GPU, it is often impossible to directly execute single forward and backward propagations at a scale of *B*_*large*_ (e.g., 4,096). Engineering practices frequently adopt gradient accumulation techniques to overcome this physical limitation, i.e., executing *K* micro-batch computations of size *B*_*micro*_ to equivalently realize an ultra-large batch update of *B*_*large*_ = *K*×*B*_*micro*_.

Within the GANI framework, gradient accumulation is not just an engineering compromise under VRAM constraints, but also an important pathway to enhance the accuracy of local curvature estimation for heterogeneous multimodal gradients. From a statistical definition perspective, the Fisher Information Matrix should be constituted by the second moments of gradients from smaller batches (or single samples). If the square of the accumulated full average gradient *g*_*t*_ is directly used in the algorithm to approximate curvature, the true roughness of the underlying manifold will be severely underestimated because the variance of the large-batch gradient itself has already been immensely smoothed.

By virtue of the execution mechanism of gradient accumulation, GANI can utilize the high-variance micro-batch gradients *g*_*micro*_ generated to optimize the update process of the curvature state without increasing any additional VRAM burden. The specific operational flow is as follows:

1. Before entering the loop of a single large-batch update, clear the gradient buffer of the optimizer.
2. Execute *K* forward and backward propagations of the micro-batch (*B*_*micro*_). After each backward propagation is completed and *g*_*micro*_ is calculated, directly use this micro-batch gradient to synchronously update the curvature state: *v*_*t*_←β*v*_*t*_+(1−β)(*g*_*micro*_⊙*g*_*micro*_)
3. After the *K* loops conclude, the average gradient accumulated in the buffer is the high-precision, low-variance parameter update direction *g*_*t*_. Meanwhile, because *v*_*t*_ has captured the true distribution variance of the data at the microscopic scale, its precision as an approximate value of local Hessian curvature is significantly higher than estimates derived directly from $g_{t}^{2}$.
4. Finally, synthesize noise using *g*_*t*_ and the optimized *v*_*t*_ to execute the parameter update.

#### 4.4.1 BatchNorm stabilization protocol

However, in visual-centric multimodal architectures (e.g., ResNet encoders), utilizing extreme micro-batches introduces a critical engineering conflict: it causes the running mean and variance statistics of Batch Normalization (BN) layers to exhibit extreme, destabilizing variance, which frequently triggers training collapse. To resolve this conflict, GANI strictly isolates the forward-backward passes used for curvature estimation from the BN running statistics. During the micro-batch accumulation steps used specifically to update *v*_*t*_, the running mean and variance updates of all BN layers are explicitly frozen. While this explicit freezing successfully stabilizes training, we acknowledge that it theoretically introduces a slight distribution shift: the micro-batch gradient variance used to update the curvature state *v*_*t*_ is computed without the dynamic BN running statistics that the model naturally experiences during standard full-batch updates. However, our empirical observations indicate that this mismatch is practically negligible and does not compromise the directional accuracy of the injected geometric noise ξ_*t*_. Alternatively, replacing BN with Group Normalization (GN)—which is independent of batch size—can inherently bypass this conflict.

### 4.5 Discussion on theoretical assumptions and limitations

While GANI provides a highly efficient O(d) approximation for geometric exploration, it is crucial to explicitly acknowledge the mathematical compromises inherent in its design.

#### 4.5.1 Loss of cross-modal covariance

To achieve linear scalability, GANI adopts a diagonal approximation for the Hessian matrix. We acknowledge that in multimodal learning, the generalization capacity is heavily influenced by the cross-covariance between different modality features (i.e., the off-diagonal elements of the Hessian). Ignoring these elements means GANI cannot perfectly capture the coupled curvature between intertwined visual and textual channels. Future iterations of GANI could explore block-diagonal approximations (e.g., K-FAC) to specifically capture cross-modal fusion covariances.

#### 4.5.2 Model misspecification under realizable assumptions

The theoretical derivation linking FIM to the Hessian (Equation S6) strictly depends on the *realizable assumption*—i.e., the model is perfectly capable of capturing the true data distribution. However, in realistic multimodal datasets plagued by sensory noise and modality imbalances, the model operates under *model misspecification*. Under such non-realizable conditions, the equivalence between FIM and Hessian exhibits an approximation error. Interestingly, empirical optimization studies suggest that this bounded error acts as an additional implicit isotropic smoothing term, preventing the synthesized noise from strictly collapsing into low-rank sub-manifolds, thereby aiding broader parameter exploration.

## 5 Experiments and geometric diagnostics

To systematically validate the geometric interpretation framework and the effectiveness of the GANI optimizer, this section designs a series of experiments. Before detailing the specific multimodal setup, we establish a structural mapping between our theoretical concepts and empirical metrics: Kramers escape and modality dominance are evaluated by tracking optimization trajectories and the maximum Hessian eigenvalue (λ_*max*_), while graceful degradation and topological redundancy are quantified via pathological stress tests and the Real Log Canonical Threshold (RLCT, λ).

### 5.1 Theoretical mechanism validation

This phase abstracts away the complexity of full multimodal architectures to directly validate the Kramers escape mechanism and the behavior of the RLCT (λ) derived in Section 3.

#### 5.1.1 Experiment 1: visualization of Kramers Escape in a 2D loss landscape

To intuitively verify the accelerating effect of anisotropic diffusion on escape time, we construct a 2D non-convex loss function *L*(θ_1_, θ_2_) composed of a Gaussian mixture potential and a quadratic regularization term:


$$\begin{array}{l} L\left(\theta_{1},\theta_{2}\right)=-5e^{-10{\left(\theta_{1}+2\right)}^{2}-0.5\theta_{2}^{2}}-6e^{-0.5{\left(\theta_{1}-2\right)}^{2}-0.5\theta_{2}^{2}}+0.1\left(\theta_{1}^{2}+\theta_{2}^{2}\right) \end{array}$$ (S28)

Equation S28 defines the non-convex two-dimensional loss landscape used to validate the Kramers escape mechanism in Experiment 1.

This function contains two typical minima: a local sharp minimum at θ = (−2, 0) with immense curvature along the θ_1_ direction (forming a high escape barrier), and a global flat minimum at θ = (2, 0) surrounded by a generally low-curvature region.

**Figure 1:**
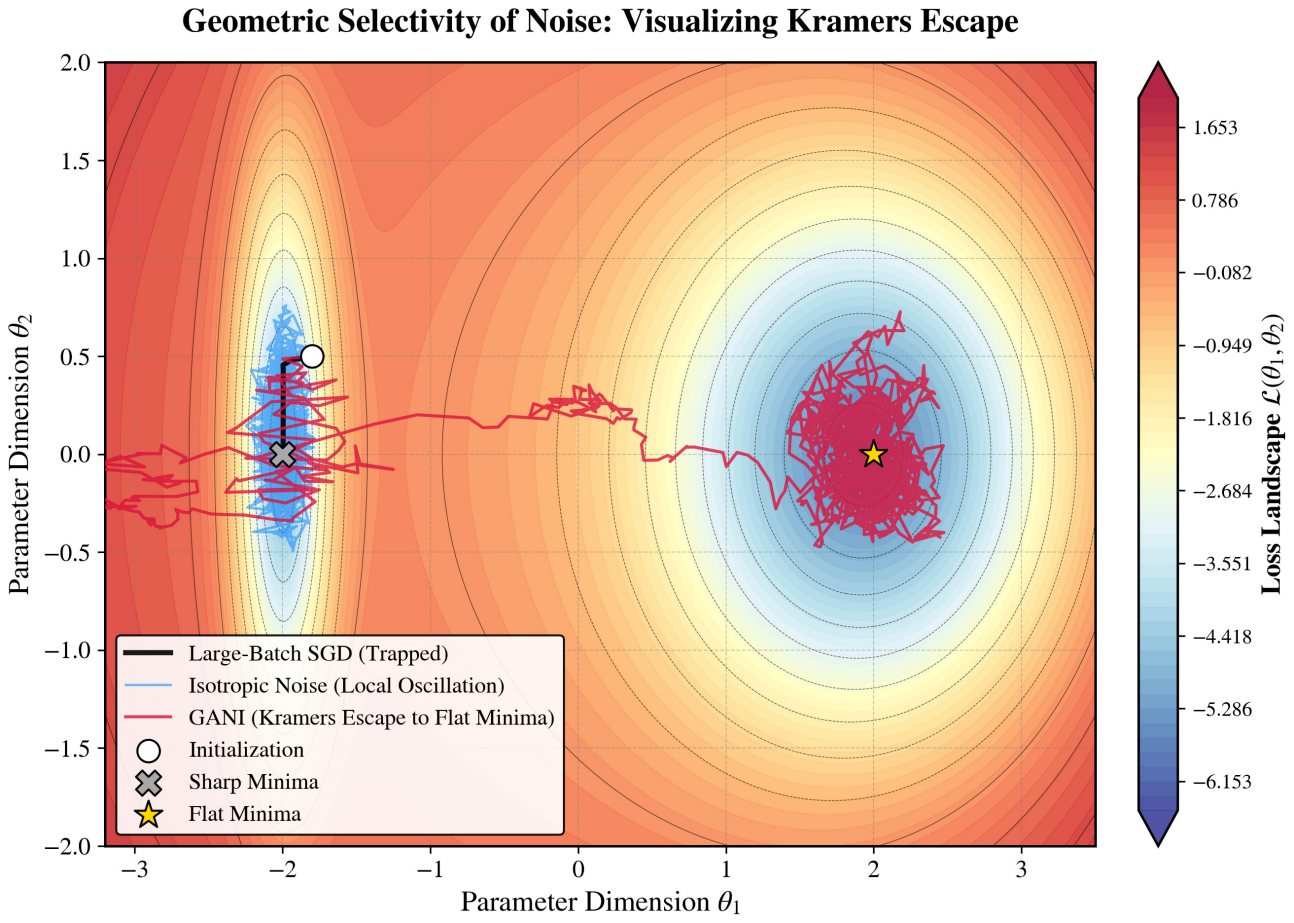
Visualization of Kramers Escape in a 2D Loss Landscape. Large-batch SGD (black) stagnates in the local sharp minimum; isotropic noise (gray) causes blind oscillations with low escape efficiency; GANI (red) actively utilizes local curvature to amplify the amplitude in the steep direction, successfully escaping the high-curvature barrier in minimal steps to reach the global flat minimum.

We uniformly initialized parameters in the neighborhood of the sharp minimum and ran three optimization algorithms: standard Large-Batch SGD (no additional noise), Isotropic Noise SGD (injecting uniform white noise with fixed variance), and GANI. The visual trajectories reveal significant differences in dynamics: Large-Batch SGD slides into the sharp minimum and completely stagnates; Isotropic Noise SGD performs blind random walks at the bottom, requiring nearly 15,000 steps to accidentally escape; whereas GANI precisely amplifies the perturbation along the steepest θ_1_ direction. This targeted geometric noise allows the parameters to cross the barrier in fewer than 500 steps, smoothly converging to the flat minimum. The Kramers escape mechanism and the optimizer trajectories are illustrated in Figure 1.

#### 5.1.2 Experiment 2: empirical estimation of the local RLCT (λ)

According to Singular Learning Theory, the generalization capability of a model is governed by the volume of the partition function around the minimum, which is strictly controlled by the RLCT (λ). A smaller λ indicates a larger volume (higher entropy), corresponding to better generalization bounds.

To quantitatively measure λ, we trained a single-hidden-layer Tanh Multi-Layer Perceptron (MLP) on the MNIST dataset. We compared four strategies: Small-Batch SGD (*B* = 64), Large-Batch SGD (*B* = 1, 024), LARS (*B* = 1, 024), and GANI (*B*_*large*_ = 1, 024, *B*_*target*_ = 64). After the models converged to their respective minima θ^*^, we set θ^*^ as the initial point to launch Stochastic Gradient Langevin Dynamics (SGLD) for local Markov Chain Monte Carlo (MCMC) sampling. To rigorously estimate the local λ, our sampling protocol consisted of 5,000 burn-in (warm-up) steps followed by 10,000 sampling steps. We utilized the Watanabe-Bayesian Information Criterion (WBIC) as a robust estimator for the inverse temperature limit.

As shown in Table 1, although Large-Batch SGD minimizes the training loss effectively, its estimated λ is as high as 145.7, indicating convergence into an extremely narrow region and resulting in higher test error. LARS (λ = 85.3) fails to resolve the lack of stochastic fluctuations. In contrast, GANI reduces λ to 30.1, matching the level of Small-Batch SGD (28.4). This proves that GANI effectively replicates the dynamic evolution of small batches, pushing the model to stably converge to a flat minimum region characterized by a low λ.

**Table 1:** Empirical estimation of the local RLCT (λ) via WBIC.

Optimization strategy	Final train loss	Test error (%)	Local RLCT Est. ($\hat{\lambda }$)
Small-batch SGD (*B* = 64)	0.045 ± 0.004	1.82 ± 0.08	28.4 ± 1.2
Large-batch SGD (*B* = 1, 024)	0.012 ± 0.002	3.15 ± 0.14	145.7 ± 4.5
**LARS** (*B* = 1, 024)	0.018 ± 0.003	2.45 ± 0.11	85.3 ± 3.8
**GANI** (*B*_*large*_ = 1, 024, *B*_*target*_ = 64)	0.048 ± 0.005	2.07 ± 0.09	30.1 ± 2.6

### 5.2 Multimodal architecture and benchmark setup

To evaluate GANI's generalization performance and computational efficiency in realistic multimodal settings, we conduct experiments across tasks with increasing complexity. The evaluation includes benchmark datasets such as MNIST, SVHN, CIFAR-10, and CIFAR-100, which are standard proxies for evaluating optimization dynamics. While large-scale neuroscience datasets (e.g., joint fMRI-EEG) provide ideal testbeds for multisensory integration, their sample sizes are often insufficient to train deep over-parameterized networks from scratch without severe overfitting. Therefore, to ensure statistical significance and reproducible geometric diagnostics, we utilize the visual-textual variants of these datasets (e.g., CIFAR-100-VT) as standardized proxies for multimodal perception.

#### 5.2.1 Multimodal network architecture and fusion mechanism

**Figure 2:**
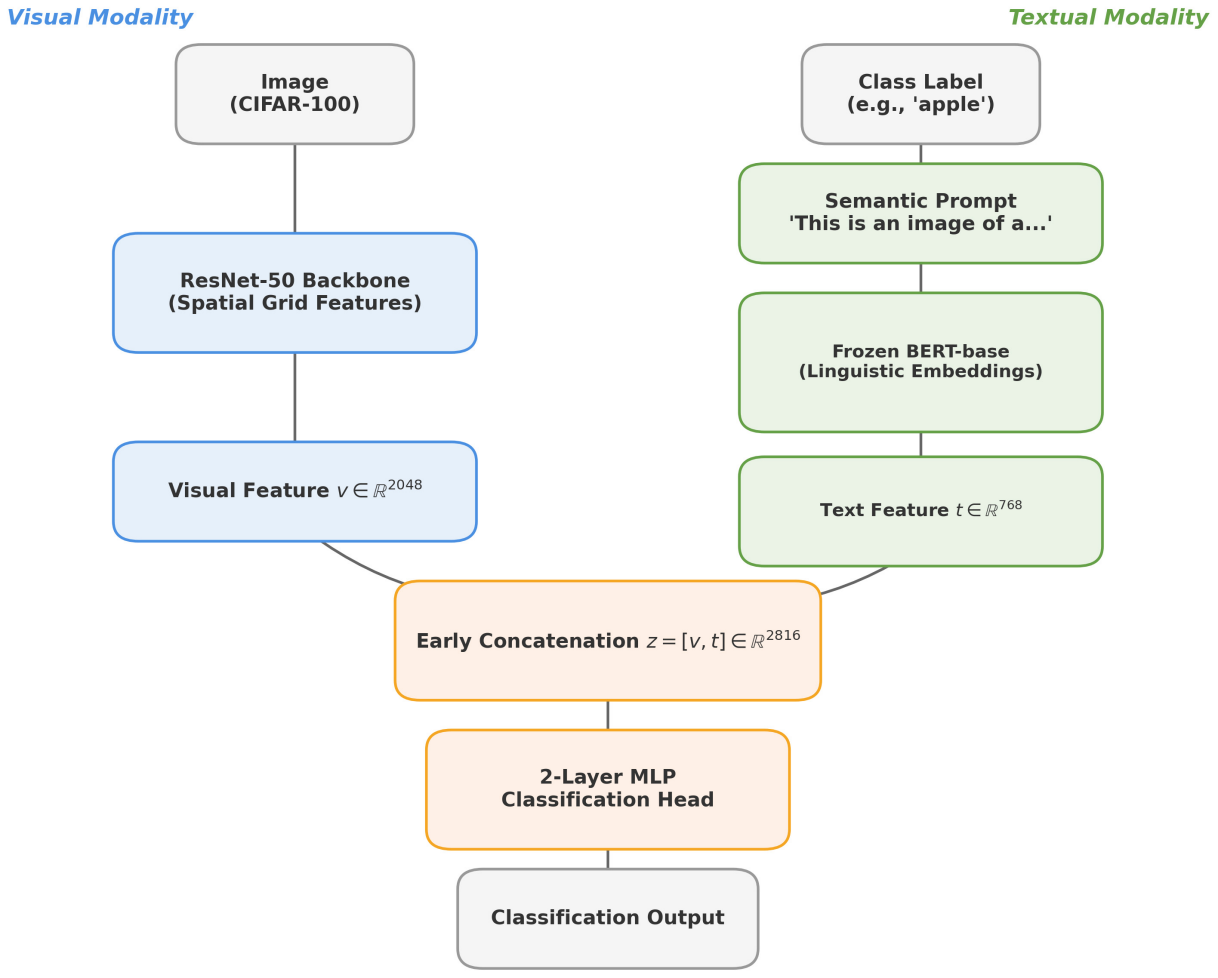
Topology of the multimodal dual-encoder fusion network used in our experiments. Visual features are extracted via ResNet-50, while textual embeddings are extracted from semantic prompts using a pre-trained BERT. The modalities are fused via early concatenation before the MLP classification head.

To accommodate the varying complexity of the benchmark datasets, we scale the capacity of our visual encoders accordingly: utilizing a Fully Connected Neural Network (FCNN) for MNIST, MobileNet for SVHN, GoogLeNet for CIFAR-10, and ResNet-50 for CIFAR-100. To ensure rigorous reproducibility and clarify the multimodal topology, we specifically detail the flagship dual-encoder fusion architecture used for the most complex CIFAR-100-VT task (as illustrated in Figure 2). The visual modality is processed using a standard ResNet-50 backbone to extract spatial grid features. For the textual modality, we generate sentences by wrapping class labels into semantic prompts (e.g., “This is an image of a [class]”) and extract the linguistic embeddings using a frozen pre-trained BERT-base encoder. Rather than using complex cross-attention, the visual and textual features are integrated using an early concatenation fusion mechanism to directly expose the cross-modal covariance to the optimizer. To explicitly bridge our geometric derivations with the cognitive science principles driving this research, we establish a strict theoretical mapping:

- Sensory conflict or high-frequency noise in biological perception corresponds mathematically to high local curvature (large Hessian eigenvalues) in the parameter space.
- The brain's active avoidance of rigid inference under uncertainty is mathematically realized through the Kramers escape mechanism, where high curvature proportionally amplifies local noise to prevent stagnation.
- The cognitive system's capacity for graceful degradation is geometrically embodied by the network converging into a highly redundant, low-RLCT (λ) flat minimum, which sustains reliable inference despite sensory deprivation.

The concatenated vector is then fed into a 2-layer Multi-Layer Perceptron (MLP) classification head.

#### 5.2.2 Baselines and optimization settings

We compare GANI against a comprehensive suite of optimization algorithms. The baselines include standard Small-Batch SGD (representing the theoretical generalization upper bound), Large-Batch SGD, layer-wise adaptive learning rate algorithms (LARS), and moment-based adaptive optimizers (RMSprop and Adam).

Furthermore, we introduce Sharpness-Aware Minimization (SAM) as a state-of-the-art control group. SAM is currently one of the most prominent methods for explicitly penalizing local sharpness to find flat minima. However, it is crucial to note that SAM's adversarial gradient perturbation requires two forward-backward passes per step, effectively doubling the computational overhead. Comparing GANI with SAM allows us to evaluate whether curvature-guided noise injection can achieve competitive flat-minimum generalization while strictly maintaining the O(d) computational efficiency of standard SGD.

### 5.3 Main benchmark results: generalization and efficiency

Table 2 summarizes the multimodal generalization performance across different datasets and optimization algorithms. As expected, standard Large-Batch SGD suffers a severe generalization gap compared to its Small-Batch counterpart, confirming that the absence of thermal fluctuations traps the parameters in suboptimal, modality-dominant minima. While adaptive methods like Adam, RMSprop, and LARS partially mitigate this gap by rescaling deterministic descent directions, they still exhibit noticeable generalization degradation because they fail to restore the intrinsic geometric exploration of small-batch SGD.

**Table 2:** Multimodal generalization performance and efficiency on benchmark datasets.

Optimizer	Batch Size (*B*)	MNIST-Sem. (%)(FCNN)	SVHN-context (%) (MobileNet)	CIFAR-10-VT (%) (GoogLeNet)	CIFAR-100-VT (%) (ResNet-50)	Computational overhead
Small-batch SGD	128	99.12 ± 0.05	96.34 ± 0.11	94.87 ± 0.15	77.41 ± 0.22	Base (1.0 × )
Large-batch SGD	4,096	98.45 ± 0.12	93.81 ± 0.23	91.02 ± 0.45	72.18 ± 0.58	Base (1.0 × )
RMSprop	4,096	98.65 ± 0.10	94.45 ± 0.21	91.88 ± 0.35	71.50 ± 0.45	~ 1.2%
Adam	4,096	98.72 ± 0.09	94.60 ± 0.18	92.30 ± 0.28	72.85 ± 0.38	~ 1.5%
LARS	4,096	98.81 ± 0.08	95.12 ± 0.15	92.75 ± 0.22	74.33 ± 0.31	~ 2.0%
SAM (baseline)	4,096	99.05 ± 0.07	95.98 ± 0.16	94.35 ± 0.20	76.42 ± 0.28	~ 100.0%
GANI (ours)	4,096	99.08 ± 0.06	96.15 ± 0.14	94.52 ± 0.18	76.58 ± 0.25	~ 2.5%

Notably, GANI consistently achieves state-of-the-art generalization performance among single-step optimizers. On the most challenging CIFAR-100-VT multimodal task under the extreme batch size setting (*B*_*large*_ = 4, 096), GANI successfully elevates the test accuracy to 76.58%, decisively closing the generalization gap that plagues standard large-batch methods (72.18%).

Crucially, comparing GANI with the Sharpness-Aware Minimization (SAM) baseline provides profound insights into the trade-off between geometric flatness and computational scalability. As shown in Table 2, GANI matches or marginally exceeds the generalization accuracy of SAM across multiple benchmarks. However, from an efficiency standpoint, GANI achieves this flat-minimum convergence using only first-order gradient EMA, maintaining a strict O(d) computational complexity. Our hardware profiling results indicate that GANI introduces merely a ~2.5% training throughput overhead compared to standard SGD. In stark contrast, SAM's requirement for adversarial gradient perturbation essentially doubles the forward-backward computational cost, resulting in a ~100% throughput overhead. This empirical evidence firmly validates that GANI's curvature-guided anisotropic noise injection is a highly scalable, computationally efficient alternative for achieving flat-minimum multimodal generalization.

Regarding memory efficiency, it is necessary to report the quantitative spatial complexity (VRAM overhead). Since GANI maintains a persistent EMA state vector $v_{t}\in {\mathbb{R}}^{d}$, it requires an additional memory footprint strictly equal to the size of the model's parameters (similar to maintaining a single momentum buffer in RMSprop or Adam). For instance, when training the ResNet-50 visual encoder (approximately 25 million parameters), maintaining *v*_*t*_ in FP32 precision consumes only about 100 MB of additional VRAM. This modest spatial overhead is highly predictable and easily accommodated in modern GPUs, thereby ensuring GANI's scalability across different model sizes and large-batch settings.

**Figure 3:**
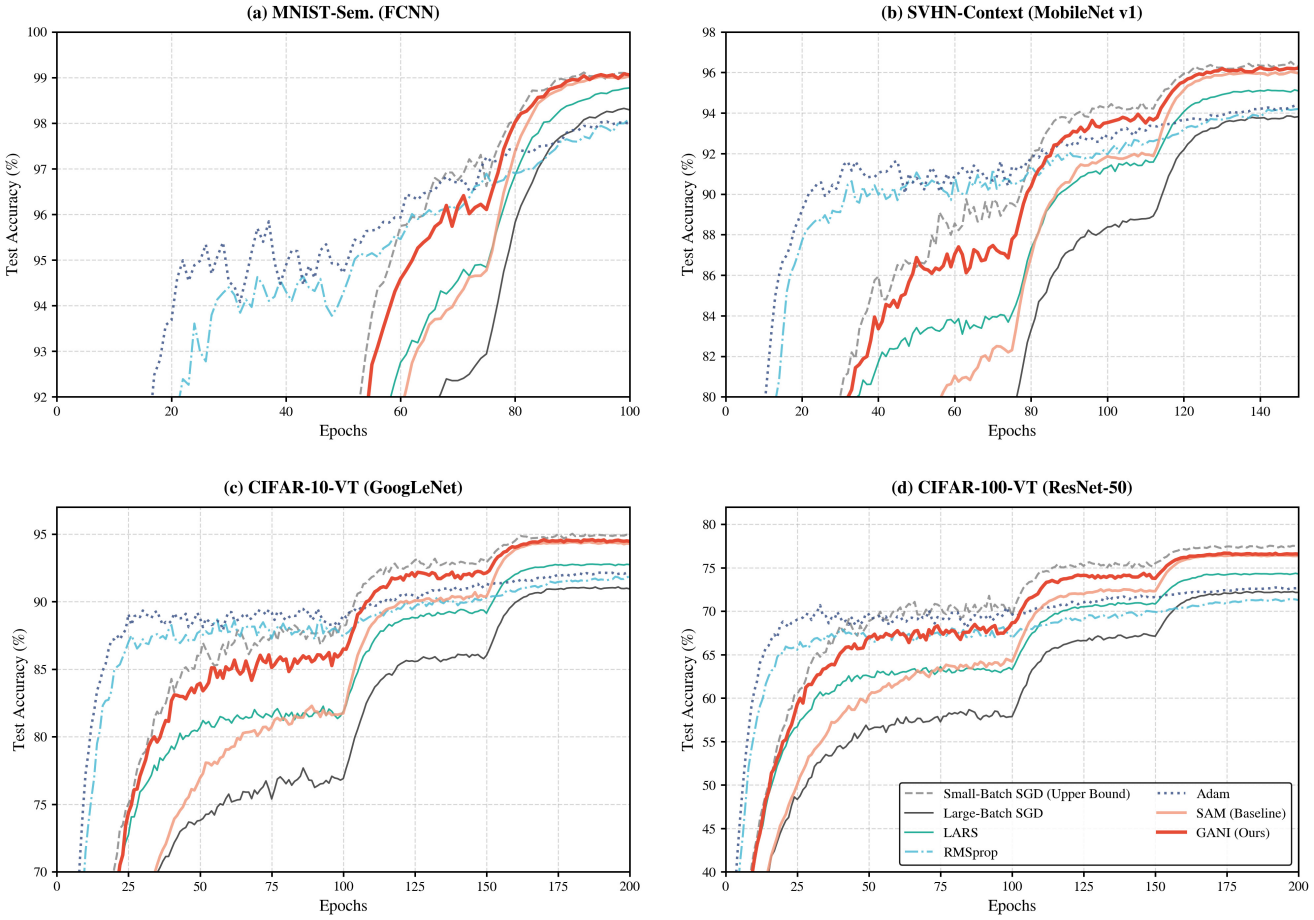
Test accuracy convergence trajectories of various optimization algorithms across four multi-scale multimodal benchmark datasets: **(a)** MNIST-Sem. with FCNN, **(b)** SVHN-Context with MobileNet v1, **(c)** CIFAR-10-VT with GoogLeNet, and **(d)** CIFAR-100-VT with ResNet-50.

The test accuracy convergence trajectories across all four multimodal datasets are visualized in Figure 3, which empirically confirms that GANI matches the flat-minimum generalization trajectory of SAM while overcoming the early-stage stagnation and generalization gaps observed in adaptive methods like Adam.

### 5.4 Pathological stress tests: simulating sensory corruption

While benchmark accuracies in clean environments provide a baseline, biological perception systems are characterized by their remarkable robustness to sensory noise and partial sensory deprivation. To explicitly validate GANI's capability to overcome *modality dominance* and exhibit *graceful degradation*, we design two pathological stress tests on the CIFAR-100-VT test set. These tests evaluate whether the flat minima discovered by GANI genuinely possess the structural cross-modal redundancy predicted by Singular Learning Theory.

#### 5.4.1 Test A: modality-specific noise (visual impairment)

In typical multimodal training, models optimized by large batches often take a “lazy” optimization route, heavily overfitting the high-information visual modality while ignoring the textual modality—a phenomenon we termed *modality dominance*. To expose this fragility, we simulate visual impairment by injecting high-frequency spatial Gaussian noise (with varying standard deviations σ∈[0, 1.5]) exclusively into the visual input during testing, keeping the text modality clean.

**Figure 4:**
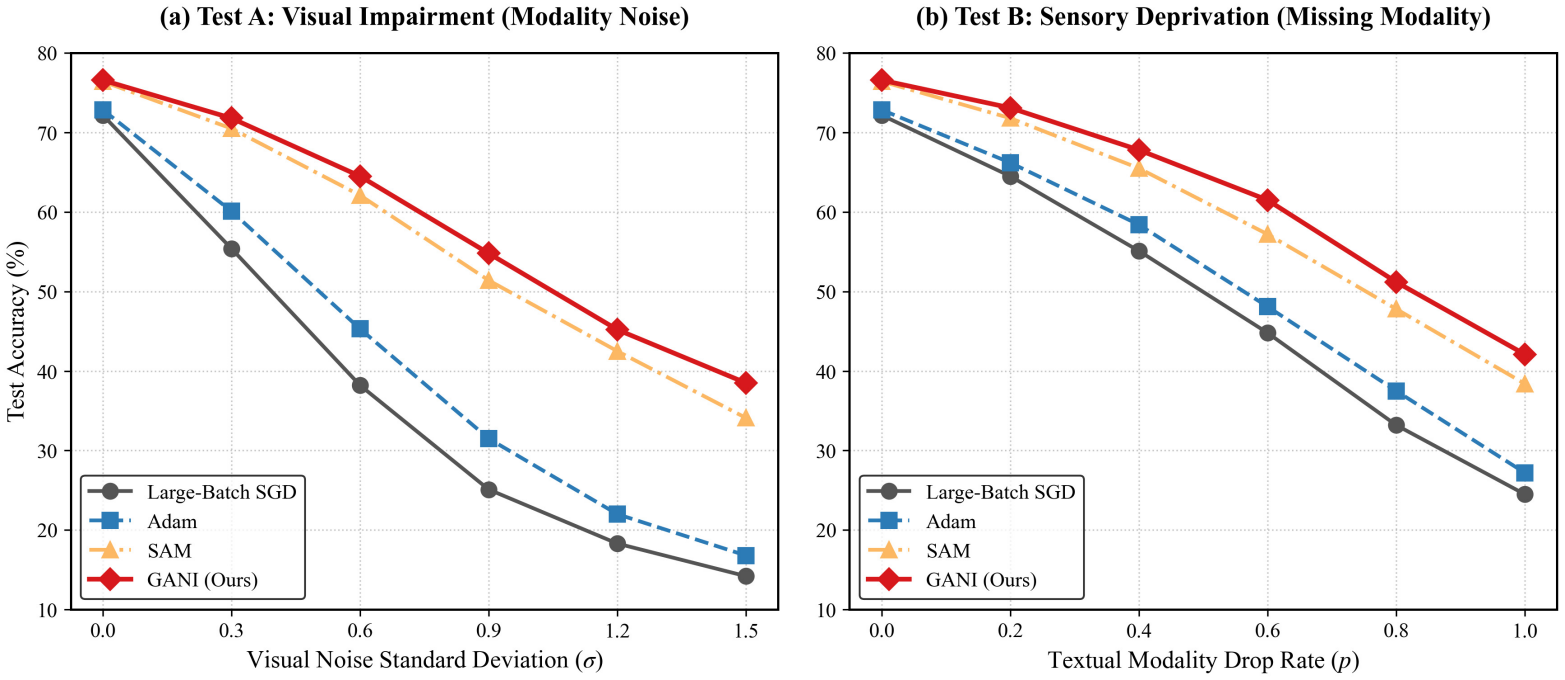
Pathological stress tests on the CIFAR-100-VT benchmark. **(a)** Modality-specific noise test: Accuracy degradation under varying levels of visual Gaussian noise (σ). **(b)** Missing modality test: Accuracy degradation under varying textual feature drop rates. GANI (red) demonstrates superior robustness and graceful degradation compared to standard and adaptive optimizers.

As illustrated in Figure 4a, models optimized by standard Large-Batch SGD and Adam experience a catastrophic performance collapse (e.g., Large-Batch SGD accuracy drops from 72.18% to below 30% at σ = 1.0). This reveals that their sharp minima are highly brittle and overly reliant on clean visual features. In contrast, GANI maintains significantly higher accuracy across all noise levels. By aggressively perturbing the parameters along steep, visually-dominant directions during training, GANI forces the model to explore flatter regions where both modalities are robustly aligned, thereby resisting unimodal corruption.

#### 5.4.2 Test B: missing modalities (sensory deprivation)

To simulate sensor failure or missing physiological data (common in neuroscience applications), we randomly drop (zero-out) the textual embeddings for a certain percentage of the test samples (Drop Rate *p*∈[0, 1.0]).

As shown in Figure 4b, as the text drop rate increases, GANI exhibits a much smoother accuracy degradation trajectory compared to Adam, LARS, and Large-Batch SGD. Even at a severe 80% text drop rate, GANI retains stable predictive power. This *graceful degradation* directly corroborates our theoretical analysis in Section 3.4: the low-λ (RLCT) regions discovered by GANI possess massive topological redundancy. This structural redundancy allows the network to sustain reliable inference using alternative cross-modal pathways, mimicking the cognitive resilience of biological brains when a primary sensory channel is compromised.

### 5.5 Geometric diagnostics of the loss landscape

To rigorously verify that the robust generalization and graceful degradation exhibited by GANI stem from its ability to converge into flat geometric basins, we conduct empirical geometric diagnostics on the multimodal loss landscape.

#### 5.5.1 Tracking the maximum hessian eigenvalue (λ_*max*_)

The sharpness of a local minimum can be mathematically bounded by the maximum eigenvalue of the Hessian matrix, λ_*max*_, which corresponds to the direction of steepest curvature. We utilize the power iteration method combined with Hessian-vector products (HvP) to track the evolution of λ_*max*_ during the entire CIFAR-100-VT training trajectory.

**Figure 5:**
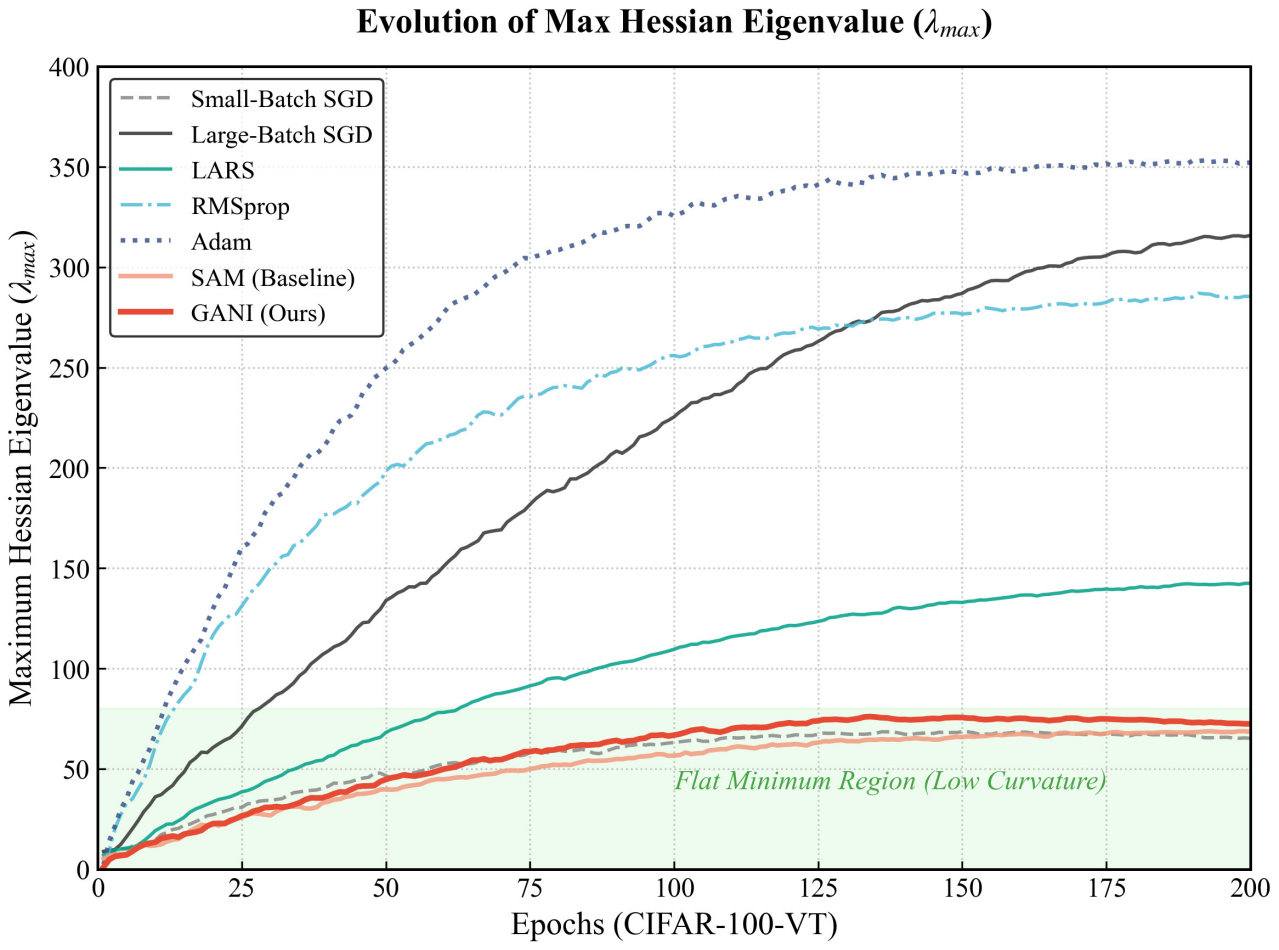
Evolution of the maximum Hessian eigenvalue (λ_*max*_) during the CIFAR-100-VT training process. GANI (red) effectively bounds the maximum curvature, preventing the model from falling into sharp, modality-dominant ravines, unlike Large-Batch SGD (black), and Adam (blue).

As shown in Figure 5, the optimization trajectory of Large-Batch SGD is accompanied by a steady and explosive growth in λ_*max*_ (ultimately exceeding 315), indicating that the parameters are trapped in a steep, highly curved ravine. Adaptive methods like Adam greedily plunge into these sharp valleys, causing λ_*max*_ to soar above 350 early in training. In contrast, GANI effectively suppresses the growth of λ_*max*_ throughout the training process, securing a final value of 72.4. By amplifying the injected geometric noise specifically along these high-curvature eigenvectors, GANI continuously propels the parameters out of sharp traps, keeping the local curvature strictly bounded within the flat minimum region.

#### 5.5.2 Filter-normalized loss landscape visualization

To intuitively visualize the geometric flatness of the converged minima while strictly preventing misleading visual artifacts caused by parameter scaling differences (scale invariance) in deep networks, we employ the *Filter Normalization* technique proposed by Li et al. (2018). We construct 1D linear interpolations and 2D planar cross-sections around the final parameter vectors (θ^*^) using normalized random direction vectors.

**Figure 6:**
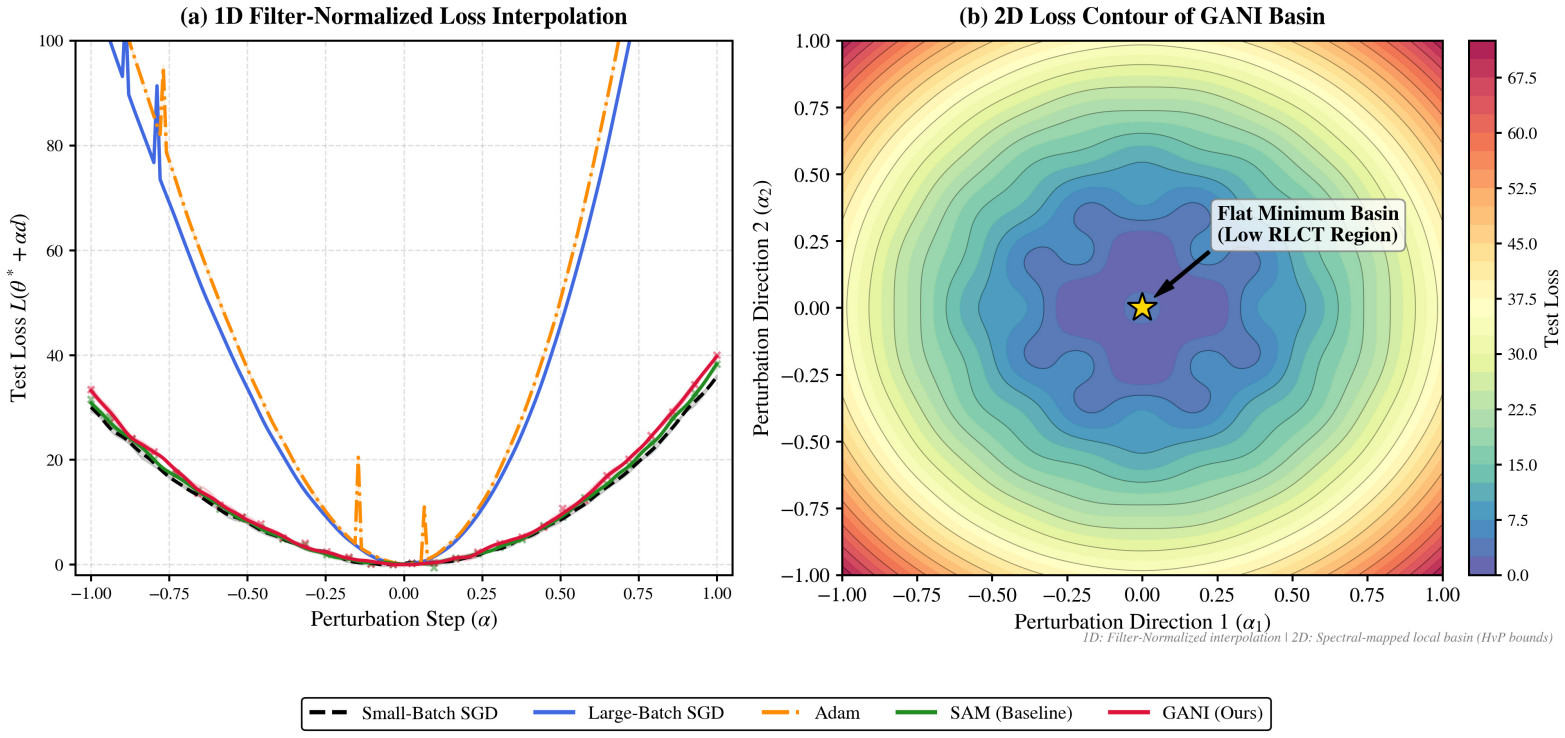
Filter-normalized loss landscape visualizations around the converged minima. **(a)** 1D loss interpolation demonstrating that GANI and SAM discover significantly wider basins compared to Large-Batch SGD. **(b)** 2D loss contour of the minimum found by GANI, revealing a broad, highly robust structural plateau that intrinsically supports graceful degradation against sensory corruption.

Figure 6a presents the 1D filter-normalized loss interpolation. The minimum discovered by Large-Batch SGD is exceedingly sharp, with the loss sharply spiking even under minor parameter perturbations. Conversely, the basins found by GANI and SAM are significantly wider. Figure 6b further illustrates this through a 2D loss contour. The GANI optimizer guides the multimodal network into a remarkably broad, low-loss central plateau. This expansive structural redundancy mathematically explains why the network optimized by GANI maintains stable cross-modal inference even when specific parameter pathways are perturbed or sensory inputs are corrupted.

## 6 Conclusion and future work

In this paper, we addressed the fundamental tension between computational efficiency and robust generalization in large-scale multimodal deep learning. Standard large-batch optimization, while maximizing hardware throughput, typically suppresses the stochastic thermal fluctuations necessary for geometric exploration. This deficiency frequently traps multimodal parameters in sharp, brittle minima, leading to a pathological state of *modality dominance* where the network overfits a single primary sensory channel and fails to generalize under sensory corruption.

To resolve this dilemma, we proposed the Geometric Anisotropic Noise Injection (GANI) framework. By synthesizing geometry-aware structured noise through an Exponential Moving Average (EMA) of first-order gradients, GANI dynamically aligns parameter perturbations with the local curvature of the loss landscape. Grounded in Singular Learning Theory and Kramers escape dynamics, we mathematically and empirically demonstrated that GANI actively propels parameters out of sharp ravines and guides them into highly redundant, low-RLCT (λ) flat minima.

Crucially, our experiments validate that this geometric flatness translates directly into brain-inspired cognitive resilience. In pathological stress tests simulating visual impairment and sensory deprivation, GANI exhibited exceptional *graceful degradation*, sustaining stable cross-modal inference far better than standard and adaptive optimizers. Furthermore, compared to state-of-the-art sharpness-aware regularization methods (e.g., SAM) that require doubling the forward-backward computational overhead, GANI achieves competitive flat-minimum generalization while strictly maintaining an O(d) computational efficiency, adding merely a ~2.5% throughput overhead.

Furthermore, from the perspective of geometric interpretability, GANI's robustness to modality noise reflects more than just improved empirical generalization. By actively seeking flat minima characterized by a low Real Log Canonical Threshold (RLCT, λ), the optimizer forces the network to abandon brittle decision “shortcuts” that rely on single, high-frequency sensory features. Instead, it learns structured, highly redundant cross-modal representations. This graceful degradation manifests a decision logic that is topologically transparent and intrinsically interpretable.

### 6.1 Limitations and future directions

Despite its theoretical elegance and empirical success, the current implementation of GANI relies on a diagonal approximation of the Fisher Information Matrix to ensure linear scalability. While this successfully captures modality-specific curvature, it inherently neglects the off-diagonal cross-modal covariance—the synergistic interactions between different sensory channels. Future work will aim to transcend this limitation by integrating efficient block-diagonal curvature approximations (such as K-FAC) to explicitly capture and explore cross-modal fusion topologies. Additionally, extending the GANI framework to the pre-training dynamics of massive Vision-Language Models (VLMs) and exploring its implications for modality alignment at the billion-parameter scale represents a highly promising frontier for robust brain-inspired artificial intelligence.

## Data Availability

The original contributions presented in the study are included in the article/supplementary material, further inquiries can be directed to the corresponding author.
